# Dynamic influences on cooperation in a social dilemma: How type of experience and communication affect behavioral spillovers

**DOI:** 10.1371/journal.pone.0213038

**Published:** 2019-03-12

**Authors:** Bart J. Bruin, Henri C. Dekker, Tom L. C. M. Groot

**Affiliations:** Department of Accounting, School of Business and Economics, Vrije Universiteit Amsterdam, Amsterdam, The Netherlands; Middlesex University, UNITED KINGDOM

## Abstract

In many work and decision situations, realizing cooperation among individuals is important. However, decision making environments of individuals are far from stable, resulting in changes in task complexity and the social settings they encounter. We argue that past experiences with cooperative behavior can result in different cooperative norms and expectations about the behavior of others and will have an effect on an individual’s subsequent behavior in new situations. This study experimentally investigates these dynamics of cooperative behavior in social dilemmas and addresses the role of communication to provide empirical evidence about a cognitive mechanism that may lead to these spillovers. Specifically, the experimental design randomly assigns subjects to one type of repetitive interactions in the first social dilemma (single partner or different partners) and we then examine how this impacts the propensity to behave cooperatively in subsequent social dilemmas with unfamiliar partners (either single or different). Because of the variety in complexity of decision-making environments in practice, we do so by examining behavioral spillovers across three different social dilemmas that vary in difficulty to make cooperation successful. Our findings show that individuals cooperate more during initial interactions with a single partner. More importantly, this has positive spillover effects for subsequent behavior and communication, even to settings without repeated interactions with a single partner. However, environmental conditions affect the ability to transfer established norms of cooperation to subsequent interactions, as an initially learned cooperative norm is gradually replaced by a more competitive attitude when individuals start to interact with unfamiliar others in a setting in which cooperative success is more difficult to achieve. Our findings illustrate the power of repeated interactions for establishing and sustaining cooperation in other settings and enhance understanding of how cooperative decisions can be shaped by both incentives and the broader behavioral context of individuals.

## Introduction

Cooperation is traditionally formed to achieve goals that cannot be achieved by an individual acting alone, and often requires individuals to efficiently combine and coordinate their resources [1–3]. Fostering cooperation between individuals whose actions have strategic interdependence can be problematic because of opportunistic behavior, especially when they do not know each other and when it is difficult to communicate intentions or enforce cooperation [4, 5]. In these social dilemmas where individual outcomes are also dependent on the decisions of others, the interdependence structure among individuals is a key factor in predicting cooperative behavior [6]. Repeated interactions with the same individual make cooperation more advantageous, while an awareness that the interaction is likely to end soon may induce uncooperative behavior in the present [7]. In many organizational instances, though, these social settings are rarely stable [2, 8, 9]. For instance, works team are often reassembled due to corporate restructuring, downsizing, voluntary career movement or reassignment of tasks [2, 10, 11], or are formed for a limited duration, such as a project team [8]. In addition, tasks often differ in complexity while changing consumer preferences, new government regulations, or new technologies can also require changes in decision practices to succeed. Sustaining cooperation over time then not only requires individuals to condition their actions on the behavior of unfamiliar others, but also to actively respond to changes in environmental conditions that require different resource coordination strategies for cooperative success. In this study we address this issue with data from a social dilemma laboratory experiment.

Dynamics in interaction settings allow for behavioral spillovers, where subsequent decisions are influenced by individuals’ prior experiences [4, 9, 12–16]. Based on interdependence theory [17] and organizational learning theory [18], we argue that the type of interaction in one social dilemma (either a single partner or different partners) impacts individuals’ propensity to behave cooperatively in subsequent social dilemmas with unfamiliar partners. We further expect the persistence of this spillover effect to depend on environmental conditions, which in our experiment are reflected by the contributions necessary to establish cooperative success. Given the variety in complexity of decision-making environments in practice, we examine cooperation spillovers across three social dilemmas that vary in difficulty of making cooperation successful. Interactions characterized by greater strategic uncertainty, reflecting a higher proportion of resources that individuals stand to lose if cooperation turns out to be unsuccessful, are more demanding on individuals’ belief formation and produce a greater cognitive load [4, 12]. We hypothesize that the extent to which individuals overcome these reservations depends on initial experience. We also address a cognitive mechanism behind this behavior. How individuals communicate with each other is expected to have an important mediating role in this process, as it provides an opportunity to assess each other’s commitment to cooperation, identify task requirements and coordinate on task interdependencies [19–21].

We implemented a 2x2 experimental design, crossing two types of initial experience (single vs. different partners) with the same two types of subsequent experience to test our expectations. Participants were randomly assigned to the treatments and within their treatment were randomly matched with another participant to create a dyad. All participants played three consecutive periods of repetitive interactions; one for initial experience and two for subsequent experience. After each period, all participants were rematched with a new partner. Participants could communicate with each other through an integrated chat box.

The main contribution of this study is to evaluate how individuals’ prior experiences and communication tactics affect behavioral spillover effects across different decision-making environments that vary in interdependence structures as well as the required decision-strategy to be successful. We find that initial repeated interactions allow for the establishment of social norms of cooperation which can be sustained with other unfamiliar individuals, even without repeated interactions. However, environmental conditions affect the ability to transfer established social norms of cooperation to subsequent interactions, as an initially learned cooperative norm is gradually replaced by a more competitive attitude when individuals start to interact with unfamiliar others in a setting in which cooperative success is more difficult to achieve. Our findings also illustrate the power of initial repeated interactions to establish and sustain cooperation over time. Once cooperative norms have been routinized in the interaction style of individuals, they seem to become more resilient to failures. Individuals are then more likely to initially devote failures to environmental conditions and increase their cooperative resource commitment, while individuals who initially interacted with different partners are more likely to misinterpret failure as opportunistic behavior of their new partner, given that they have experienced this before. Together, these results show how cooperative decisions can be shaped by both incentives and the broader behavioral context of individuals and enhance understanding of the persistence of cooperative spillover effects across different settings.

Our results also have practical relevance as they can create awareness for managers about spillover effects in the assignment of work activities to employees. Considering the sequences of interaction settings helps to understand how assignment of tasks with varying complexity and interdependence structures could be designed in order to improve performance.

## Theoretical background

### Uncertainties in social dilemmas

The conflict between competition and cooperation in interactions between interdependent parties has been extensively examined through the lens of a social dilemma [22]. The key issue surrounding social dilemmas is that individuals must pool resources to create synergistic shared benefits, but once produced, these benefits can be enjoyed by everyone regardless of their contribution level [23, 24]. Van Lange et al. ([25], p. 126) define the resulting social dilemma as: “a situation in which a non-cooperative action is (at times) tempting for each individual in that it yields superior (often short-term) outcomes for self, and if all pursue this non-cooperative course of action, all are (often in the long-term) worse off than if all had cooperated”. Highest collective and individual outcomes often occur when all partners choose to cooperate, the dilemma is that for cooperative behavior to make sense individuals must believe that the partner will cooperate as well [1, 24, 26–30]. Social dilemmas have frequently been used to model situations within society at large that involve interdependent actions [22] (for reviews see [25, 31]), but they can also model behavior in business contexts, both within firms and between firms. For example, several studies [6, 19, 32] show that interfirm relationships, such as alliances or joint-ventures, can be considered as social dilemmas. These collaborations can provide a competitive advantage compared to other resource investments, but also present several opportunities to behave opportunistically, the primary reason for collaborative failures [33].

In a review of the literature about the effects of uncertainties on behavior in social dilemmas, Van Dijk et al. [34] distinguish two types of common uncertainties in real world social dilemmas: uncertainty about whether partners will cooperate and uncertainties about characteristics of the dilemma itself. Uncertainty about partners’ cooperative intentions refers to the possibility that a partner is not committed to mutual interests [35], behavioral or social uncertainty [2]. Uncertainties regarding the characteristics of the dilemma refer to environmental uncertainties. Van Dijk et al. [34] consider the so-called provision threshold, the required investment for successful cooperative activities, a primary environmental issue. This corresponds to real settings where the synergistic potential of collaboration cannot be specified in advance [6, 25]. Prior studies have shown that behavioral and environmental uncertainty independently reduce the willingness to cooperate [34]. Others conjecture that behavioral and environmental uncertainty influence each other [36, 37]. For instance, individuals’ estimates about others’ cooperativeness depends on the level of environmental uncertainty [29]. Hence, individuals need to resolve these uncertainties for efficient coordination of resources and cooperative success.

### Prior studies on behavioral spillovers

No behavior exists in a vacuum, and decisions are often influenced by individuals’ prior experiences [4, 12–16, 38–41]. This transfer of behavior is referred to as behavioral spillover. Behavior can spill over when tasks are performed simultaneously [12, 42], or sequentially [4, 15, 38, 41, 43] and can have positive or negative effects on subsequent performance (for a review, see [14]). Different types of learning can be the source of behavioral spillover. Individuals can learn about the structural properties of their decision environment and transfer this to the context they subsequently face or they can learn about the social preferences of others and refer to these behavioral heuristics in subsequent interactions [4, 12, 16, 38, 41, 43, 44].

Experience from previous interactions has been shown to have an effect on future play in other games, but most studies focus on the same groups playing related games in sequence [38, 41, 43, 45]. Another collection of studies investigates behavioral spillovers across different environments, but not multiple social dilemmas [2, 4, 12]. As such, there is limited research on what factors cause behavioral spillovers to occur across related settings when individuals need to interact with unfamiliar others. Studies have shown that transfer of behavior generally occurs in strategic interaction settings that are descriptively similar [4, 15, 39]. In addition, decision-making environments in practice are far from stable. Individuals interact in many different settings, sometimes repetitively with the same individual and sometimes with other individuals, resulting in changing interdependence structures over time [8, 11]. In addition, tasks often differ in complexity while changing consumer preferences, new government regulations, or new technologies can also require changes in decision practices to succeed. We thus contribute to this literature on behavioral spillovers by considering behaviors that take place sequentially and are structurally linked by the same underlying task principle of individuals, however characterized by different interdependence structures and difficulty in being successful (e.g. different provision thresholds). Specifically, we examine how individuals respond to a new interdependence structure and required changes in decision practices to succeed and whether their behavior differs depending on their prior interdependence structure. We examine this expected difference in behavior informed by different types of experience by an outcome-based measure, performance, as recommended by Argote and Miron-Spektor [46]. In traditional social dilemma research, the term cooperation is used to reflect choices favoring collective interests over individual interests [22, 32]. In our study, we focus on how individuals figure out the details of efficiently implementing cooperation with partners. Cooperative choices in social dilemmas can be inefficient and costly for individuals when the partner does not contribute (sufficiently) to cooperation, while free-riding on a partner’s cooperative contributions can be efficient and beneficial for individuals. We therefore do not focus on cooperative choices but on individual performance (e.g. payoffs).

### Interdependence structure

Organizational learning theory considers experience as fundamental for learning [18]. Argote and Miron-Spektor [46] argue that learning from experience interacts with context to create knowledge. Knowledge acquired through learning is embedded in the context and this affects future learning and behavior [4, 12, 47]. In social dilemmas, the context is primarily created by the features of the decision-making environment, in other words the interdependence of decisions that determine outcomes. Interdependency theory describes how individuals can affect another’s outcomes during the course of an interaction [17]. Since social dilemmas tempt individuals to behave non-cooperatively in the short-term with the consequence of being worse off in the long-term, the awareness of possible future encounters can have a strong influence on their behavior [6, 25]. Concerns about future consequences (e.g. the shadow of the future) can affect whether individuals choose to maximize the long-term or short-term consequences of their actions [16, 26, 30, 44, 48–51] and whether they trust the cooperative intentions of others [21]. Hence, single interactions or repeated interactions can impact the willingness to cooperate and the ability to familiarize and learn from others.

Several studies on individual learning have examined individual outcomes based on two different work-design related strategies: specialization and variety [52–54]. These studies show that specialization allows to capture the benefits of repetition while variety in task content is considered to keep individuals more motivated and provides them with more opportunities to learn. The potential gains of motivation and learning attributed to heterogeneous experiences are considered to be especially relevant for the long term when conditions change, as variety in prior experiences increase the likelihood that new situations can be related to what is already known [55]. However, studies have shown that switching tasks initially worsens individual performance because of cognitive switching costs [54, 56]. We do not examine a variety of tasks but expect variety in task context (e.g. interdependence) to have a similar effect. Multiple single interactions create heterogeneous experience with different individuals, while repeated interactions induce more homogenous experience. During interdependent decision-making, learning is not just individual learning about advantages and disadvantages of cooperative and non-cooperative behavior, which can simply be transferred to interactions with other individuals. Cooperation must be enacted and sustained in the interaction with others. When only one individual understands the benefits of cooperation, the outcome can be disastrous for that individual as cooperating also bears the greatest risks [32]. During interactions with different partners, there is a continuous need to assess cooperative intentions of the other party. This continuous suspicion of opportunistic behavior breeds distrust and decreases confidence in cooperative success in the future, even in the absence of opportunistic intentions by any of the partners [44, 47, 57]. Consequently, we expect that the learning potential of interactions with different partners is hard to materialize without prior experience how to cooperate. Repeated interactions with the same partner provide a stable relationship where the value of future interactions provides a long-term orientation [44, 49]. This increases reciprocal decisions between individuals and facilitates the development of trust and cooperative norms between individuals [15, 21]. We thus hypothesize:

#### Hypothesis 1a (H1a)

In repetitive interdependent decisions, initial interactions with a single partner result in higher performance than initial interactions with different partners.

### Communication

Simply observing outcomes of choice behavior does not reveal the cognitive mechanisms behind this behavior [31]. Cooperation not only exists when individuals’ goals are related or better aligned, but also because they coordinate and work together to achieve these goals. Individuals need to learn how to assess each other’s commitment to cooperation, identify task requirements and coordinate on task interdependencies. Communication provides the opportunity to share this knowledge, as it allows to highlight shared interests and common goals, recognize and share opportunities for improvement, make commitments about own behavior or demonstrate cooperative intent [19–21, 58, 59]. Hence, we examine how communication content mediates the effect of interdependence structure on performance. Most studies that examine how communication content influences cooperative behavior distinguish distributive and integrative communication [58]. In our study, we label these communication tactics as non-cooperative and cooperative. Non-cooperative communication is focused on maximizing individual outcomes, while cooperative communication seeks to establish coordination through accurate information exchange with the intent to realize the interests of both partners. The relative extent of cooperative communication will thus positively affect cooperative behavior. However, when there is a higher likelihood that the other party uses shared information for its own benefit instead of for the collective, individuals are less likely to reveal their cooperative intentions or communicate truthfully [21] and less likely to attend to information they receive [60]. Together, this reduces the possibility to create consensus and integrate perspectives. On the contrary, repeated interactions provide a motivation to build a reputation for honestly revealing intentions and private information, thus making communication efficiency enhancing [61].

#### Hypothesis 1b (H1b)

The effect of initial interdependence structure on performance is positively mediated by cooperative communication.

### Lower threshold value and subsequent interdependence

A new interdependence structure, with an unfamiliar individual, can make individuals less willing to contribute to the cooperation without previous evidence of the other party’s cooperative willingness [62]. We hypothesize that overcoming these initial reservations is dependent on initial experience. With initial experience from interactions with a single partner, individuals have learned how to cooperate efficiently [4]. Studies show that repetitive interactions with a single partner result in establishing a cooperative norm, which does not emerge when interacting with different partners [16, 38, 44]. However, Duffy and Ochs [44] also report a dramatic decline in cooperative behavior of individuals after a switch from fixed pairing to random matching. On the other hand, Cason et al. [4] and Peysakhovich and Rand [16] show that individuals’ belief formation and choice behavior in new strategic situations with other individuals is based on norms formed in similar past situations. In addition, Engle-Warnick and Slonim [63] show that individuals are more likely to start cooperating in a new group after a long than after a short initial match. Cooperative commitment and norms from prior interactions thus can lower perceived uncertainty of the new environment and promote adaptive behavior [4, 15]. We expect that individuals who have learned to cooperate with a single partner can develop a social norm of cooperation that can persist with others, because of the lower threshold value during subsequent interactions. Their prior cooperative routines are still successful, which confirms their expectation of cooperative intentions of their new partners. In addition, they learn that the lower threshold value reduces their exposure risk (i.e. the proportion of the total amount of resources that individuals stand to lose if the cooperation turns out to be unsuccessful) which further increases the prospect of cooperative benefits [49]. During subsequent interactions with a lower threshold value, we expect that individuals who initially interacted with a single partner are not likely to shift from a cooperative norm to a competitive attitude when interacting repetitively with different partners.

Initial experience with different partners creates skepticism about intentions of others and elicits a more competitive norm, which makes it difficult to foresee value in cooperative interactions [44, 63]. As such, they are less likely to learn that the exposure risk is significantly lower, which further increases their initial competitive orientation [47]. However, when individuals start to interact repetitively with a single partner after initial interactions with different partners, this should lower the perceived risk of opportunistic behavior by the partner and they will be more inclined toward cooperation compared to individuals that continue to interact with different partners [49]. Zeng and Chen [32] argue that the shift from short-term to long-term thinking occurs when individuals realize the importance of cooperation based on an understanding of the advantages and disadvantages of cooperative and non-cooperative behavior. This is underlined by Duffy and Ochs [44], who show that a norm of non-cooperation after interacting with different partners is directly replaced with a cooperative one after a switch to a single partner. Recognition of the value of successful cooperation increases the willingness to further align cooperative decisions with their new partner. As such, we expect that individuals who initially interacted with different partners need time to adapt to an environment where the risk of opportunistic behavior is lower but will do this better and faster when they have repetitive interactions with a single partner than with different partners.

#### Hypothesis 2a (H2a)

During subsequent repetitive interactions with a lower threshold value, initial and/or subsequent interactions with a single partner lead to higher performance than initial and subsequent interactions with different partners.

A social norm of (non-)cooperation can be a precedent that increases or decreases an individual’s subsequent cooperative choices [4, 15, 41, 44], but a cooperative norm does not always increase cooperation [32]. A new decision environment requires sharing information and processing of information in order to adapt behavior and decisions. We thus hypothesize that cooperative communication mediates the behavioral spillover effect.

#### Hypothesis 2b (H2b)

The behavioral spillover effect of initial experience on subsequent performance during interactions with a lower threshold value is mediated by cooperative communication.

### Higher threshold value and subsequent interdependence

Interactions characterized by more strategic uncertainty are more demanding on individuals’ belief formation and produce greater cognitive load [4, 12]. This reduces the likelihood that individuals contribute to the cooperation, out of fear that others will not contribute [29]. A higher threshold value will hence initially increase the likelihood of cooperative failures. We expect that the attribution of this cooperative failure will differ across individuals, depending on their initial experience. Individuals mitigate uncertainty in their decision environment via information on how referent others acted before. Hyndman et al. [21] show that fixed pairings create opportunities for learning and adjustment, but individuals are just as likely to learn that they are a bad match for each other. Individuals who initially interacted with different partners are more likely to misinterpret failure as opportunistic behavior of their new partner, given that they have experienced this before [59, 64]. Cooperative failure re-activates their initial competitive mindset and the likelihood that they classify their new partner as untrustworthy. They will start protect themselves from being exploited at the expense of finding a mutual solution to adapt to the new environment. Individuals that have experienced consistent cooperative intentions of partners develop a social norm of cooperation [44]. Cooperation becomes more routinized as a decision heuristic, which makes it more resilient to failures [5]. They will initially devote failures to environmental conditions and increase their cooperative resource commitment. Performance of individuals interacting with a single partner during subsequent interactions with a higher threshold value is thus expected to be higher when they have initial experience from interactions with a single partner than with different partners. We also expect that this hypothesized effect is mediated by communication.

For individuals who interact with different partners during subsequent interactions with a higher threshold value, we expect no performance difference based on their initial experience. Bereby-Meyer and Roth [47] show that in a complex environment, behavior becomes more dependent on what actions others are choosing. A higher threshold value increases the risk of cooperation failure and makes the exposure risk more pronounced [29]. It will become difficult to maintain a social norm of cooperation that persists when interacting with different partners in a more competitive setting [44]. Individuals will lose confidence in cooperative benefits, infer that partners are increasingly pursuing self-interests, and consequently will become more competitive themselves.

#### Hypothesis 3a (H3a)

During subsequent repetitive interactions with a higher threshold value, initial and subsequent interactions with a single partner lead to higher performance than initial and/or subsequent interactions with different partners.

#### Hypothesis 3b (H3b)

The behavioral spillover effect of initial experience on subsequent performance during interactions with a higher threshold value is mediated by cooperative communication.

## Method

### Ethics statement

This study and its consent procedure were approved by the Research Ethics Review Board of the Vrije Universiteit’s School of Business and Economics. Informed verbal consent from the participants was taken prior to data collection and all participants were debriefed. The verbal consent procedure involved informing the participants about the nature, purpose and intended use of the results of the study. Verbal consent was considered to be sufficient, since it was ensured that data were stored and analyzed anonymously. All subjects were given a unique anonymous identification number for privacy protection. In addition, the experiment was non-invasive and participation was voluntary, with the opportunity to decide whether or not to take part in the study.

### Experimental game

We use a social dilemma game, in which cooperation is modeled as a public goods assurance dilemma. Assurance dilemmas are unique because highest collective and individual outcomes occur when both partners choose to cooperate [25, 31]. This correspondence of joint and own outcomes might suggest that the solution is simple and hence there is no dilemma. However, in assurance public goods dilemmas all members must contribute or the public good is not produced [29, 65]. The dilemma is that for contributing to the cooperation (public good) to make sense, individuals must believe that the others will cooperate as well [32, 66]. In addition, public goods dilemmas provide a temptation to under-contribute toward the public good and free-ride on contributions of others, as once produced, the benefits of public goods can be enjoyed by everyone regardless of contribution level [24]. For this, we use a threshold value (provision point) such that cooperation is only successful (e.g. the public good provided) if voluntary contributions exceed the required threshold level of contributions [29, 66]. This threshold value is higher than the amount of resources under control of an individual, supporting the principle that successful cooperation requires investments of both partners, but does not require investment of an individual’s full amount of resources. This specifically tempts individuals to exploit others’ cooperative efforts by free riding on their investments. We further increased the tension between competitive and cooperative behavior by allowing individuals to obtain private benefits from their partners’ cooperative resource investment when cooperation turned out to be unsuccessful (total cooperative resource investment below provision point). This incentive quantifies that cooperative relationships also provide an opportunity to gain private benefits once acquired knowledge, resources or skills of partners are leveraged in areas unrelated to the cooperation [6].

Each participant in the experiment played the role of a firm manager making decisions about the allocation of resources with the objective to create profits for their firm. The participants decided on allocating their controlled resources towards individual production activities, which would generate profits privately to them, and towards cooperative production activities, which could generate profits for them and their partner. For each allocation decision (each round within a game) they had to allocate the full amount of their controlled resources, but they had full discretion in how they allocated resources. Participants had two and a half minutes for each round to make their decision. Supporting information ‘S1 Appendix’ provides a full copy of the experimental task instructions provided to the participants.

To induce proper incentives that represent a threshold assurance dilemma, the profits for the participants within a dyad were calculated as follows. For every decision round, both players received a fixed amount of 150 resources to invest. For each resource invested in individual production activities, a player received 1.000 Experimental Euros (E€) of profit, which were private profits. To make cooperative activities successful, a minimum amount of resources, the threshold value, was required. Participants were not informed about the value of this threshold in any game. If total investment in cooperative activities was above the threshold value, the partners received a fixed bonus of 500.000 E€. This bonus was equally shared between both players (250.000 E€ each) regardless of their respective resource investments. All resources invested above the threshold yielded an additional total profit of 200 E€ (100 E€ for each partner) per resource. Investments in cooperative production activities below the threshold did not yield any cooperative profits (i.e. zero bonus and no common return per unit of resource invested). Players received 500 E€ private profit per resource invested in unsuccessful cooperative production activities by their partner. The threshold values were set at 198 resources in game one, 158 in game two and 238 in game three. This sequence of threshold values refers to the sequence in our hypotheses. A higher (lower) threshold value requires a higher (lower) cooperative resource commitment by partners, making cooperative investments more (less) risky and cooperative success more (less) difficult to achieve. To further clarify the tensions between cooperation and non-cooperation that are created with these incentives, supporting information ‘S2 Appendix’ provides more detail about the payoffs that could be obtained with a variety of investment outcomes (e.g. full cooperative contribution, no cooperative contribution, contributing exactly the threshold) given the incentives and specific game characteristics.

Participants received feedback about their own investment decision, whether the cooperation was successful, their own profit from individual production activities and total round profit, and their own cumulative profit for the respective game. To avoid reputational effects, we did not provide participants with actual investment decisions or profits of their partners in prior rounds or games. Participants received information regarding the last round played and were not able to review information from prior rounds. With this information participants could learn how to adapt their resource allocations. When cooperation failed, they could calculate exactly how much their partner invested in cooperative production activities by subtracting their individual production activities profit from their total round profit. After successful cooperation, participants were able to calculate the total overinvestment in cooperative production activities, by subtracting their individual production activities profit and share of the fixed bonus from their total round profit. In both scenarios (failure and success), participants could not individually calculate the exact threshold value. For this, they needed to share accurate and reliable information with each other about their resource investment decisions and profits. The integrated chat box provided the opportunity to do this.

### Experimental design and treatments

The entire experiment was computerized, using a web-application developed for this experiment. The experiment was divided into three games and a game consisted of a fixed number of seven rounds. To avoid endgame effects participants were informed that the experimental session constituted a series of games with each game divided into a number of rounds. Within a session, each game started at the same time for all participants and they all played the three games sequentially in the same order. Each round within a game started at the same time for both partners in the dyad, and decisions within a dyad were made simultaneously.

We implemented a 2x2 experimental design, crossing two types of initial experience with two types of subsequent experience. Participants were randomly assigned to the treatments and within a treatment were randomly matched with another participant to create a dyad. We varied the matching protocol to create dyads wherein participants interacted repeatedly with the same partner or had repetitive interactions with different partners. Before the start of each game, participants were informed that they would be matched with the same partner in every round of a game or that they would be matched with a partner for one round and paired with another partner each subsequent round. The interaction mode in game one determined initial experience of participants. Subsequent experience is represented by the second and third game. Half of the participants continued interacting in the same type of interaction mode, the other half changed from single partner interaction mode to different partners interaction mode or vice versa. This switch in matching protocol was not announced in advance. Each participant, irrespective of treatment, was matched with a new partner at the start of each new game. During each round of a game, participants were allowed to communicate with each other.

### Procedures and participants

One hundred and sixty-six (166) students participated in one of five sessions (number of participants per session: 46 (1); 20 (2); 30 (3); 32 (4); 38 (5)). Each session lasted approximately 75 minutes and each subject participated in only one session. All students were recruited from the Accounting and Control program at a large Dutch university. We recruited both third-year Bachelor students as well as Master students. The majority of students were male (118 male; 48 female) and most students were enrolled in the full-time program (128 full-time; 38 part-time). Gender and educational background (Bachelor vs. Master) did not have any significant effects on the outcome variables and are excluded from further analyses. Part-time students participated in a separate session (5). Part-time Master students in our experiment work four days a week as an accountant or controller. Their experiences and day-to-day activities differ from those of full-time students which can influence their behavior in the experiment as well. We control for performance difference in further analyses by including a dummy variable for part-time students. Participants earned a show up fee (€ 2.25) with additional compensation based on their performance during the experiment. At the end of the experimental session, experiment euro’s earned were converted to real euro’s at the rate of E€ 1 = € 0.000001. Total compensation was paid in cash directly after the experimental session (on average €6.89, min = €4.63, max = €8.40).

Participants arrived at the computer lab and were randomly assigned to computer terminals that were divided with cubicles, to prohibit face-to-face interaction between participants. Brief general instructions were read aloud, all further instructions were provided on the computer screens once participants logged in on the web-application. Hard-copy instructions were provided as well.

As we focus on individual participants in our experiment, individual-level properties can affect their decision-making as well. We examine a setting where participants can make decisions that are risk-free (individual production investment) or risky (cooperative production investment) and the task provides participants with an incentive to free ride on the investment of partners. Prior studies show that individuals have different attitudes to risks [67] and social motives [68, 69]. In order to control for these individual differences, participants started with two questionnaires to assess their risk-aversion and social value orientation using instruments adopted from Holt and Laury [67] and Van Lange et al. [68]. Untabulated analyses show that scores on these variables do not significantly differ across the treatment groups and results of the statistical analyses that control for these individual attributes do not affect the results of our hypothesis testing. We excluded these variables from further analyses. To exclude framing effects of the instruments used to measure risk attitude and social value orientation on the main task, participants completed a distraction task (formulating words based on a string of letters) before they were provided with information about the experimental task.

Instructions first explained the decisions participants were asked to make and potential payoffs. Then the main screen of the application (where to enter allocation decisions, find outcomes from prior interactions and how to communicate with the partner) was presented and explained. Before the start of their task, each participant had to answer questions to test their comprehension on different aspects of the task. An incorrect answer on any of the questions directed participants to explanatory computer screens, to ensure understanding of the relevant aspects and payoff calculations of the task. After completing this comprehension quiz, manipulations were introduced and the first game started. Manipulation check questions asked participants after each game to indicate if they were matched with a single partner or with different partners during the last game played. Respectively 16 (9.6%), 11 (6.6%) and 16 (9.6%) participants failed to correctly answer this question for game one, two and three. Five participants failed on more than one of these questions, which we considered as a manipulation check failure. These participants were excluded from further analyses, however their inclusion did not affect the results of our hypotheses testing. To ensure that participants could not be held up indefinitely by others, there was a time limit for each round. Participants had two and a half minutes in each round to make their resource investment decision. When participants failed to invest within that time, the computer automatically filed an investment of 0 in both individual and cooperative production. One participant was excluded for the analyses of game one and game two and one was excluded for the analyses of only game one, as these participants failed to invest in more than 3 of the 7 rounds within a game, indicating a lack of understanding how to perform their task in the particular games only or insufficient attention in these games. Their inclusion did not affect the results of our hypothesis testing. At the end of the experimental session, participants completed an exit questionnaire.

### Variable definitions

Traditionally, the term cooperation is used in social dilemma studies to reflect choices favoring collective over individual interests [22]. In our study, we focus on how individuals figure out the details of implementing cooperation. The characteristics of the game imply that contributing to cooperation can be very inefficient and costly for individuals when the partner does not contribute to cooperation, while free-riding on a partner’s cooperative contributions can be efficient and beneficial for individuals. In addition, if individuals contribute all their resources to cooperation, they are worse off than if both contribute just at the threshold amount. As such, we focus on individual payoffs as our dependent variable, measured as a participant’s total profit divided by the cooperative optimal profit. We standardize profits to make them more comparable across games as the varying threshold values for successful cooperation per game determine the potential maximum profit. We define cooperative optimal profit as the profit obtained when the threshold value for successful cooperation is exactly invested through equal resource investments. This standardization is considered appropriate as input equality increases the sustainability of cooperation. Equity theory predicts that individuals will attempt to restore equity when they perceive being treated unfairly or unequally [70], leading to conflicts, less commitment to cooperation and lower cooperative profits. Reported standardized profits reflect the percentage of realized cooperative optimal profits.

The independent variables include the indicator variables for each of the two treatments. Initial experience is a dummy variable, with a value of 0 for participants that have a single partner in every round of game one and a value of 1 for participants with a different partner in every round in game one. Subsequent experience is a dummy variable, with a value of 0 for participants that have a single partner during game two and a new but single partner during game three and a value of 1 for participants that have different partners within game two and three.

We measure communication as the proportion of cooperative tactics used within a game by dividing the number of coded cooperative tactics with the sum of cooperative and non-cooperative tactics coded in a game. We focus on individual decision-making in interdependent interactions and how this behavior is influenced by the communication individuals use and hence choose the individual participant as the unit of analysis. We coded all messages that participants exchanged with each other through the chat box using a coding scheme. We examine a setting where individuals only need to coordinate on their investment in either individual or cooperative production activities, whereas most studies that code communication provide a setting where partners have to negotiate on multiple issues. The definitions of our cooperative and non-cooperative codes are developed based on prior studies that have coded communication into distributive and integrative behavior categories [71], but were adapted for the specific characteristics of our single item coordination task into cooperative and non-cooperative categories. Communication can be used for assessing the willingness of partners and self-disclosure of willingness to cooperate [19–21]. De Dreu et al. [71] argue that coordination agreements that increase profits require that partners integrate each other’s interests and perspectives, which involves the exchange of accurate information. Communication can further enhance profits when used to coordinate on investments and when it creates the necessary social capital since partners interact in an environment with non-binding agreements. We build on these four different communication objects (willingness to cooperate, information exchange, coordinating decision, and creating atmosphere) and developed an equal number of cooperative and non-cooperative tactics within these categories (total of 20 codes, see Table 1).

**Table 1:** Communication coding scheme.

Object	Cooperative tactics	Non-cooperative tactics
**Willingness to cooperate**	Request cooperation **(RC)** Self-disclosure cooperative intentions **(SDI)**	Refusal to cooperate **(RFC)** Putdowns **(PD)**
**Information exchange**	Request for information **(RI)** Provision of information **(PI)**	Refusal share information **(RFI)** Lies about information **(LI)**
**Coordinating** **decision**	Request for proposal **(RP)** Proposal **(PROP)** Prediction positive rewards **(PPR)** Promise **(PRO)**	Counterproposal **(CPROP)** Persuasive argument **(PA)** Prediction unpleasant consequence **(UC)** Lies about decision **(LD)**
**Creating atmosphere**	Positive result reaction **(PRR)** Positive reactions **(PR)**	Negative result reaction **(NRR)** Negative reactions **(NR)**
	**Neutral tactics**	
**Miscellaneous**	(e.g. off-task talk, attempts to identify partner or confusion about game characteristics **(N)**)	

We coded neutral tactics separately, to account for the statements exchanged between partners that are unlikely to have a directional influence, such as off-task talk. We also coded ‘greetings’ and ‘request for proposal’ as a separate code but excluded these codes from further analyses on communication tactics used. A request for proposal asks a partner to propose how to invest the resources, but it is impossible to assess if and how participants use this as a tactic to influence the behavior of the partner. The first author and three research assistants that were blind to the conditions and hypotheses of this study performed coding independently. We calculated Cohen’s Kappa to assess inter-rater reliability. Inter-rater agreements varied between 0.739 and 0.805, which we consider sufficient. The first author produced a single set of codes for each participant in which all disagreements between coders were resolved. The analyses on communication tactics is based on this last set of codes. We compressed approximately 13.115 messages into 8.433 codes (including request for proposal and greetings), and coded 5.298 cooperative, 1.555 non-cooperative, and 292 neutral tactics.

We also examine differences in perceptions of partner behavior to assess if these converge with our theoretical arguments. After each game, participants were asked to assess the behavior of their partner(s) on a 7-point scale (1 competitive; 7 cooperative). We report this variable as perception of partner behavior and use it to examine if this differs across treatment conditions. We further validated our content-based coding of communication with the perception of partner behavior variable. For each participant we calculated the proportion of cooperative communication used by its partner(s) in a particular game and related this to the assessments of partner behavior provided by the participants. If participants had different partners within a game, we first calculated the proportion of cooperative communication used by the partners per round and then used the average for the game. Correlations between the two measures are positive and significant for the three games (0.445; 0.499; 0.463), supporting validity of the coding-based measurement.

Finally, participants had a maximum amount of time per round to make their decisions, and thus had to coordinate decisions with their partners in a timely fashion. We measured the amount of time for decision-making as the sum of time (in seconds) used in all seven rounds within a game to assess efficiency of decision making.

## Results

### Descriptive statistics and correlations

Descriptive statistics and correlations presented in Table 2 indicate an increase in average game profits in game two (0.817 vs. 0.654) and a subsequent decrease in game three (0.730 vs. 0.817). The lowest threshold value for successful cooperation in game two explains this pattern. Average profits in game three are higher than in game one (0.730 vs. 0.654; t-statistic = 4.606, p = 0.000), despite the higher threshold value, indicating an overall task learning effect. The averages of proportion of cooperative communication are relatively stable over the three games, 0.735, 0.792 and 0.776. Proportion of cooperative communication is positively correlated with game profits (0.422 game one; 0.421 game two; 0.354 game three), indicating that participants using more cooperative communication realized higher profits. Inefficiency in decision making is associated with lower profits as shown by the correlations between time and profit for the respective games (-0.077; -0.222; -0.348). Perception of cooperative partner behavior correlates positively with game profit (0.564; 0.546; 0.629), and negatively with time (-0.094; -0.189; -0.226), indicating that perceived cooperativeness of partners makes decision making more profitable and efficient.

**Table 2:** Descriptive statistics and correlations.

		1	2	3	4	5	6	7	8	9	10	11	12
1	Game I profit		0.145	0.128	**0.480**	-0.071	-0.012	-0.025	-0.097	-0.131	**0.568**	0.018	0.072
2	Game II profit	**0.173**		**0.283**	**0.298**	**0.380**	**0.218**	-0.024	**-0.208**	**-0.218**	**0.180**	**0.446**	**0.237**
3	Game III profit	**0.159**	**0.272**		**0.269**	**0.278**	**0.447**	-0.063	-0.111	**-0.320**	**0.262**	**0.381**	**0.662**
4	Prop. coop. com. game I	**0.422**	**0.384**	**0.308**		**0.317**	**0.321**	**-0.173**	**-0.160**	**-0.131**	**0.425**	**0.205**	**0.197**
5	Prop. coop. com. game II	-0.030	**0.421**	**0.247**	**0.397**		**0.619**	-0.093	**-0.231**	**-0.207**	0.117	**0.471**	**0.299**
6	Prop. coop. com. game III	0.051	**0.262**	**0.354**	**0.373**	**0.642**		**-0.173**	-0.096	**-0.203**	**0.158**	**0.360**	**0.399**
7	Time game I	-0.077	-0.038	-0.076	-0.015	-0.038	-0.114		**0.405**	**0.396**	-0.134	-0.103	**-0.162**
8	Time game II	-0.130	**-0.222**	-0.117	-0.114	-0.147	-0.060	**0.404**		**0.559**	-0.027	**-0.261**	**-0.158**
9	Time game III	**-0.160**	**-0.200**	**-0.348**	-0.120	-0.150	-0.150	**0.373**	**0.574**		-0.090	**-0.276**	**-0.283**
10	Perception partner game I	**0.564**	**0.182**	**0.260**	**0.413**	**0.161**	**0.189**	-0.094	-0.038	-0.096		**0.213**	**0.260**
11	Perception partner game II	0.019	**0.546**	**0.339**	**0.268**	**0.543**	**0.395**	-0.100	**-0.189**	**-0.223**	0.143		**0.574**
12	Perception partner game III	0.069	**0.210**	**0.629**	**0.194**	**0.315**	**0.383**	**-0.158**	-0.131	**-0.226**	**0.236**	**0.565**	
	Mean	0.654	0.817	0.730	0.735	0.792	0.776	579.20	514.80	426.71	5.37	5.66	5.23
	Std. deviation	0.154	0.163	0.166	0.237	0.227	0.226	164.04	171.50	148.49	1.95	1.93	1.93
	Min.	0.237	0.134	0.154	0.000	0.000	0.000	126	159	117	1	1	1
	Max.	0.990	1.100	1.036	1.000	1.000	1.000	1024	1009	777	7	7	7

Pearson correlation coefficient below the diagonal and Spearman’s Rho above the diagonal. Significant correlations (p<0.05) are displayed bold.

### Hypothesis testing

Hypothesis 1a posits that initial repetitive interactions with a single partner result in higher performance than initial repetitive interactions with different partners. We used standardized profit per round as the dependent variable, partnering condition as the between-subjects factor and round (7 rounds per game) as the within-subjects factor in our hypothesis tests. We tested hypothesis 1a based on the results of game one only. Table 3 provides accompanying descriptive statistics on the round profits per treatment group (Panel A) and the results of the repeated measures ANOVA (Panel B). During initial repetitive transactions, interactions with a single partner have a beneficial effect on profits (0.696 vs. 0.616) and this effect is statistically significant (F = 11.159, p = 0.001). This provides support for hypothesis 1a. Participants that interact with a single partner also communicate more cooperatively (0.775 vs. 0.699, p = 0.037), perceive their partner as more cooperative (6.09 vs. 4.71, p = 0.000) and use less time to make their decisions (524.75 vs. 629.06, p = 0.000). This underlines that interactions with a single partner are more trustworthy, and provide an opportunity to coordinate faster and more efficiently. Panel B reveals a significant main effect of round (F = 24.066, p = 0.000), but no interaction effect between round and partnering condition (F = 1.630, p = 0.138).

**Table 3:** Results on profits for partnering treatments game I.

**Panel A–Descriptive statistics**					
	Initial experience				
Round	Single partner (n = 76)			Different partners (n = 83)	
1	0,461			0,398	
2	0,569			0,591	
3	0,704			0,577	
4	0,733			0,609	
5	0,765			0,630	
6	0,775			0,747	
7	0,863			0,762	
Standardized game profit	0,696 (0,158)			0,616 (0,141)	
Proportion cooperative communication	0,775 (0,258)			0,699 (0,211)	
Perception behavior partner	6,09 (1,643)			4,71 (1,990)	
Time used for decision making	524,75 (171,54)			629,06 (140,26)	
**Panel B–Repeated measures ANOVA on Game I profits**					
	*Sum of Squares*	*df*	*Mean Square*	*F*	*p*
*Between Subjects*					
Initial experience	1,751	1	1,751	11,159	0,001
Error	24,476	156	0,157		
*Within Subjects effects*					
Round	12,416	5,820	2,133	24,066	0,000
Round x Initial experience	0,841	5,820	0,145	1,630	0,138
Error (rounds)	80,484	907,916			
*Covariate*					
Part_time	0,033	1	0,033	0,208	0,649

In Panel A, the cells contain information regarding the number of individuals per condition (n), the average scores, and (standard deviation). Two-tailed p-values are reported in Panel B.

We used structural equation modeling to test for mediation effects of communication content. Structural equation modeling provides the opportunity to simultaneously estimate direct and indirect effects, which is impossible with ANOVA. We used SPSS AMOS with maximum likelihood estimation and bootstrap resampling (5000) to estimate the significance of the indirect effects. We report direct, indirect and total effects of our variables of interest. In the mediation analyses we use the average profit across the rounds in the game per subject as the dependent variable and consider this as one independent observation. This is a standard practice in management and organization sciences [2].

Mediation results for game one reported in Panel A of Table 4, show that the total effect of initial experience on profits (-0.079, p = 0.002, CI:[-0.126;-0.033]) is partly mediated by the proportion of cooperative communication, indicated by the significant indirect effect (-0.020, p = 0.028, CI:[-0.042;-0.002]). This shows that the negative effect of interactions with different partners is partly explained by a lower proportion of cooperative communication used, and provides support for hypothesis 1b.

**Table 4:** Mediation analyses.

**Panel A–SEM direct, indirect and total effects Game I**														
Variables							Model I							
							Prop_coop_com *Direct effect*		Profit *Direct effect*		Profit *Indirect effect*		Profit *Total effect*	
							coef.	p	coef.	p	coef.	p	coef.	p
Initial experience							-0.076	0.038	-0.060	0.008	-0.020	0.028	-0.079	0.002
Prop_coop_com									0.260	0.000			0.260	0.000
**Panel B–SEM direct, indirect and total effects Game II**														
Variables			Model II											
			Perception behavior partner game I *Direct effect*		Prop_coop_com *Direct effect*		Prop_coop_com *Indirect effect*		Profit *Direct effect*		Profit *Indirect effect*		Profit *Total effect*	
			coef.	p	coef.	p	coef.	p	coef.	p	coef.	p	coef.	p
Initial experience			-1.649	0.001	0.031	0.490	-0.022	0.120	0.004	0.859	-0.007	0.601	-0.003	0.993
Subsequent experience			-0.123	0.783	-0.011	0.844	-0.002	0.548	0.026	0.296	-0.003	0.687	0.023	0.389
Initial experience x Subsequent experience			0.526	0.402	-0.198	0.003	0.007	0.245	-0.120	0.007	-0.034	0.050	-0.154	0.000
Perception behavior partner game I					0.013	0.165			0.005	0.421	0.003	0.081	0.008	0.245
Prop_coop_com									0.191	0.009			0.191	0.009
**Panel C–SEM direct, indirect and total effects Game III**														
Variables	Model III													
	Perception behavior partner game I *Direct effect*		Perception behavior partner game II *Direct effect*		Prop_coop_com *Direct effect*		Prop_coop_com *Indirect effect*		Profit *Direct effect*		Profit *Indirect effect*		Profit *Total effect*	
	coef.	p	coef.	p	coef.	p	coef.	p	coef.	p	coef.	p	coef.	p
Initial experience	-1.649	0.001	0.307	0.298	0.021	0.651	-0.018	0.382	-0.038	0.308	-0.021	0.170	-0.059	0.117
Subsequent experience	-0.123	0.783	-1.021	0.005	0.054	0.218	-0.047	0.005	-0.079	0.015	-0.009	0.509	-0.088	0.006
Initial experience x Subsequent experience	0.526	0.402	-0.971	0.053	-0.046	0.455	-0.032	0.186	0.026	0.602	-0.013	0.445	0.014	0.804
Perception behavior partner game I			0.120	0.116	0.014	0.152	0.005	0.083	0.014	0.060	0.004	0.032	0.018	0.017
Perception behavior partner game II					0.043	0.001			0.008	0.267	0.007	0.003	0.015	0.027
Prop_coop_com									0.156	0.005			0.156	0.005

The fit indices for Model 1 are χ^2^/df (0.006/1) = 0.006, CFI = 1.00, NFI = 1.00, and RMSEA = 0.00. The fit indices for Model 2 are χ^2^/df (0.253/3) = 0.084, CFI = 1.00, NFI = 0.99, and RMSEA = 0.00. The fit indices for Model 3 are χ^2^/df (0.253/3) = 0.084, CFI = 1.000, NFI = 0.999, and RMSEA = 0.00. Cell statistics are the unstandardized coefficient estimates and two-tailed p-values. We used 5000 Maximum Likelihood (ML) Bootstraps to calculate 95% bias-corrected confidence intervals. All three models include the covariate Part-time.

Table 5 reports the results for game two. We find a significant interaction effect between initial experience and subsequent experience, indicating that the type of prior experience influences participants’ performance in subsequent interactions (F = 11.978, p = 0.001). We performed post hoc pairwise comparisons to test for simple main effects and provide additional results on the effect proposed in hypothesis 2a (see Panel C of Table 5). These results show that participants who initially interacted with different partners and continue with different partners in game two perform significantly lower than all three other treatment groups, while there are no significant differences in profit between the other three treatment groups. During subsequent interactions with a lower threshold value, participants who have learned how to cooperate with a single partner during their prior experience are thus able to also exploit this capability with unfamiliar partners. An explanation for how they exploit this capability is their use of cooperative communication, which is comparable to the communication used by participants that continue to interact with a single partner (0.846 vs. 0.834). Our results also show that a new relationship with a single partner creates recognition of the benefits of being cooperative, which seems to mitigate skepticism about cooperative intentions of partners that could have been develop during initial interactions with different partners.

**Table 5:** Results on profits for partnering treatments game II.

**Panel A–Descriptive statistics**					
	Initial experience				
	Single partner		Different partners		
	Subsequent experience				
Round	Single partner (n = 35)	Different partners (n = 42)	Single partner (n = 41)	Different partners (n = 42)	
8	0,789	0,800	0,785	0,683	
9	0,773	0,815	0,820	0,694	
10	0,786	0,921	0,761	0,627	
11	0,890	0,860	0,826	0,721	
12	0,862	0,918	0,852	0,788	
13	0,903	0,873	0,934	0,680	
14	0,940	0,903	0,930	0,782	
Standardized game profit	0,849 (0,176)	0,870 (0,104)	0,844 (0,151)	0,711 (0,169)	
Proportion cooperative communication	0,846 (0,216)	0,834 (0,191)	0,852 (0,182)	0,647 (0,251)	
Perception behavior partner	6,40 (1,459)	5,36 (1,923)	6,49 (0,978)	4,52 (2,144)	
Time used for decision making	382,34 (152,94)	560,45 (126,39)	503,34 (192,10)	590,71 (139,79)	
**Panel B–Repeated measures ANOVA on Game II profits**					
	*Sum of Squares*	*df*	*Mean Square*	*F*	*p*
*Between Subjects*					
Initial experience	1,820	1	1,820	12,777	0,000
Subsequent experience	0,770	1	0,770	5,405	0,021
Initial experience x Subsequent experience	1,706	1	1,706	11,978	0,001
Error	22,082	155			
*Within Subjects effects*					
Round	1,571	5,714	0,275	4,487	0,000
Round x Initial experience	0,371	5,714	0,065	1,060	0,384
Round x Subsequent experience	0,601	5,714	0,105	1,716	0,118
Round x Initial experience x Subsequent experience	0,291	5,714	0,051	0,830	0,542
Error (rounds)	54,277	885,619			
*Covariate*					
Part_time	2,999	1	2,999	21,051	0,000
**Panel C—Simple effects test (Bonferroni adjusted) on Game II profits**					
Initial experience	Subsequent experience	Subsequent experience	Mean difference (p-value)		
Single partner	Single partner (0,849)	Different partners (0,870)	-0,021 (0,547)		
Different partners	Single partner (0,844)	Different partners (0,711)	0,133 (0,000)		
Subsequent experience	Initial experience	Initial experience	Mean difference		
Single partner	Single partner (0,849)	Different partners (0,844)	0,005 (0,886)		
Different partners	Single partner (0,870)	Different partners (0,711)	0,159 (0,000)		

In Panel A, the cells contain information regarding the number of individuals per condition (n), the average scores, and (standard deviation). The p-values reported in Panel B and Panel C are two-tailed.

For game two we examine the mediating effects of both communication content and the perception of partner behavior in the prior game, as additional test of our theoretical arguments (Panel B, Table 4). We argued that if prior experiences are considered as (non) cooperative, this can have positive (negative) spillover effects to subsequent interactions. As such, behavior in game two can be conditioned on how a participant perceived the prior partner’s behavior. We find that perceived cooperativeness of partners in the first game has an indirect positive effect on profit, which is significant at the 10% level (0.003, p = 0.081, CI: [0.000; 0.009].

The interaction between initial and subsequent experience has a significant effect on proportion of cooperative communication (-0.198, p = 0.003, CI:[-0.326;-0.069]) and profit (-0.154, p = 0.000, CI:[-0.247;-0.069]). This effect on profit is partially mediated by proportion of cooperative communication (-0.034, p = 0.050, CI:[-0.099;0.000]). The significantly lower profits of participants with initial and subsequent interactions with different partners are partly explained by their lower use of cooperative communication. Together, these results support hypothesis 2b.

In game three we do not find a significant interaction between participants’ initial experience and subsequent experience. The main effects for initial experience (F = 4.966, p = 0.027) and subsequent experience (F = 11.351, p = 0.001) are significant (Table 6, Panel B). The results show that participants interacting with a single partner perform significantly better than participants who interact with different partners (marginal means 0.776 vs. 0.692), and participants who initially interacted with a single partner perform better than participants who initially interacted with different partners (marginal means 0.761 vs. 0.707). Given that ANOVA lacks power for detecting an ordinal interaction [72], we performed an additional one-way ANOVA with standardized game profits as dependent variable and treatment group (4-levels) as between-subjects factor and performed post hoc analyses with Bonferroni correction to test for significance of pairwise comparisons. In this analysis we again use the average profit across the rounds in the game per subject as one independent observation.

**Table 6:** Results on profits for partnering treatments game III.

**Panel A–Descriptive statistics**					
	Initial experience				
	Single partner		Different partners		
	Subsequent experience				
Round	Single partner (n = 35)	Different partners (n = 42)	Single partner (n = 41)	Different partners (n = 42)	
15	0,569	0,530	0,593	0,499	
16	0,727	0,655	0,667	0,609	
17	0,863	0,754	0,641	0,655	
18	0,755	0,698	0,776	0,637	
19	0,929	0,779	0,836	0,754	
20	0,931	0,791	0,842	0,766	
21	0,876	0,803	0,861	0,761	
Standardized game profit	0,807 (0,157)	0,716 (0,149)	0,745 (0,189)	0,669 (0,142)	
Proportion cooperative communication	0,792 (0,223)	0,798 (0,204)	0,793 (0,254)	0,723 (0,223)	
Perception behavior partner	6,07 (1,420)	4,77 (2,093)	5,65 (1,865)	4,58 (1,905)	
Time used for decision making	354,29 (145,19)	443,52 (136,43)	408,76 (146,50)	486,37 (140,40)	
**Panel B–Repeated measures ANOVA on Game III profits**					
	*Sum of Squares*	*df*	*Mean Square*	*F*	*p*
*Between Subjects*					
Initial experience	0,799	1	0,799	4,966	0,027
Subsequent experience	1,827	1	1,827	11,351	0,001
Initial experience x Subsequent experience	0,010	1	0,010	0,060	0,807
Error	25,112	156	0,161		
*Within Subjects effects*					
Round	9,479	5,953	1,592	19,969	0,000
Round x Initial experience	0,626	5,953	0,105	1,318	0,246
Round x Subsequent experience	0,157	5,953	0,026	0,332	0,919
Round x Initial experience x Subsequent experience	0,330	5,953	0,055	0,694	0,653
Error (rounds)	74,054	928,656			
*Covariate*					
Part_time	3,093	1	3,093	19,214	0,000
**Panel C—Post-hoc comparison (Bonferroni adjusted) on Game III profits**					
Partnering group		Partnering group			
Initial experience	Subsequent experience	Initial experience	Subsequent experience	Mean difference (p-value)	
Single partner	Single partner	Single partner	Different partners	0,0915 (0,082)	
		Different partners	Same partner	0,0618 (0,575)	
		Different partners	Different partners	0,1384 (0,001)	
Different partners	Single partner	Single partner	Different partners	0,0297 (1,000)	
		Different partners	Different partners	0,0766 (0,180)	
Single partner	Different partners	Different partners	Different partners	0,0489 (1,000)	

In Panel A, the cells contain information regarding the number of individuals per condition (n), the average scores, and (standard deviation). The p-values reported in Panel B and Panel C are two-tailed.

Results in Panel C of Table 6 show that participants who interacted with different partners in each game have the worst performance (0.669), however their profits in this game are not significantly lower compared to participants who had initial interactions with a single partner and subsequent interactions with different partners (0.716). This implies that an initially learned cooperative routine is gradually replaced by a more competitive attitude and participants start to protect themselves against opportunistic behavior of partners when transaction conditions increase exposure risks. Participants who interacted with a single partner during each game, perform significantly better than participants in both treatment groups of subsequently different partners (0.807 vs. 0.716, p = 0.082; 0.807 vs. 0.669, p = 0.001). If participants initially interacted with different partners and subsequently with a single partner, they do not perform significantly better than both treatment groups of subsequently different partners (0.745 vs. 0.716, p = 1.000; 0.745 vs. 0.669, p = 0.180). This result is in line with our expectation in hypothesis 3a and can be explained by how fast participants in these treatment groups adapt their investment in cooperation. While participants who interacted with a single partner during each game start their first round of the game with an average cooperative investment of 107 (profit of 0.569), they increase this to 117 in the two following rounds, resulting in average profits of 0.727 and 0.863 and even higher in later rounds. Participants who initially interacted with different partners and subsequently with a single partner however start with an average cooperative investment of 106 (profit of 0.593) and this remains relatively stable (110 and 107) in the two following rounds. One of the reasons for this can be that participants who interacted with a single partner during each game have cooperative perceptions about partners and therefore blame cooperative failure to the characteristics of the new environment. On the other hand, participants who had variety in initial experiences are more likely to blame failure to opportunistic behavior of their partner as their initial experience created skepticism about cooperative intentions of partners. Although the proportion of cooperative communication has a positive and significant influence on profits in game three (total effect 0.156, p = 0.005, CI:[0.048;0.286]), we do not find a significant direct effect of either the type of initial and subsequent partnering experience on the proportion of cooperative communication, nor an indirect effect on profit. The same applies for the interaction effect. We do find that perceived cooperativeness of the partner in game one has a significant total effect on profit in game 3 (0.018, p = 0.017, CI:[0.003;0.033]), with a significant direct and indirect effect. This also supports our argument that perceived behavior of prior partners can be decisive for how individuals attribute cooperation outcomes in subsequent interactions and their willingness to increase cooperative commitment when transaction conditions increase exposure risks.

## Conclusion

Decision-making environments that individuals encounter in practice are far from stable. Interdependence structures between individuals change over time, caused by corporate restructuring, downsizing, voluntary career movement or reassignment of tasks. In addition, tasks often differ in complexity while changing consumer preferences, new government regulations, or new technologies can also require changes in decision practices to succeed. The ability to take what has been learned and apply it in a related context with other individuals is thus an integral aspect of individual learning and behavioral spillovers. We consider this sequential behavior and specifically examine how individuals respond to respectively a lower provision threshold and a higher provision threshold when they encounter a new interdependence structure and whether their behavior differs depending on their prior interdependence structure. The experimental results support our thesis that the type of experience individuals have matters for how they respond to changing circumstances in subsequent interdependent interactions with unfamiliar individuals and hence affects their ability to efficiently respond to dynamics in their decision-making environment.

### Theoretical implications

Our findings contribute to the literature on individual learning and behavioral spillovers. Our findings suggest that the ability to develop a norm of cooperation with unfamiliar partners is path dependent and influenced by environmental conditions. Once cooperative norms have been routinized in the interaction style of individuals, they seem to become more resilient to shocks. If partners do not have any prior relationship together but use the cooperative routines they both have established before, a failure is more easily forgiven and blamed to the encountered conditions of the task environment. Prior experience thus allows for the establishment of social norms of cooperation which can subsequently mitigate behavioral uncertainty with other individuals. However, environmental conditions affect the ability to transfer established social norms of cooperation to subsequent interactions. A change to a context of higher behavioral uncertainty does not affect performance of individuals that learned to cooperate with someone else when it is less difficult to achieve cooperative success (and individuals thus have a low exposure risk), but an initially learned cooperative norm is gradually replaced by a more competitive attitude when behavioral uncertainty is high and individuals interact in a setting that makes cooperative success more difficult to achieve (and thus imply high exposure risks). Together, these results show how cooperative decisions can be shaped by both incentives and the broader behavioral context of individuals and enhance understanding of the persistence of cooperative spillover effects across different settings. This identification of contextual conditions that support the realization of the potential value of experience moves forward the specification of when different types and combinations of experience have positive or negative effects on learning outcomes in both the short and longer term. This can further offer prescriptions on how to structure organizational work activities, both within and between firms.

### Practical implications

Cooperation is a central concern for many managers. Our results can create awareness for managers about spillover effects in the assignment of work activities to employees. The order of settings that individuals encounter is often a choice variable, as organizations can structure the work environment of their employees. Considering the sequences of interaction settings helps to understand how assignment of tasks with varying complexity and interdependence structures could be designed in order to improve performance. Our results could also have practical implications for managers responsible for joint ventures or alliances, who are concerned about being exploited by partners. The key characteristic of collaborative partnerships centers on the cooperation of individuals across firm boundaries. Casting these cooperative relationships as social dilemmas, our results highlight the importance of initial interaction structures between individuals for managing fears of exploitation by others over time.

### Limitations and future research

This study is subject to several limitations that provide opportunities for future research. First, experiments enable to test the impact of specific mechanisms while controlling for factors that confound with these mechanisms in real-world settings. But a laboratory is also more abstract and less realistic than a real-world situation. For instance, deceptive behavior may be easier when communication is not face-to-face, as in our experiment. On the other hand, this may also signal that greater reliance on technology-mediated communication can increase uncertainty about intentions and behavior of others, and as such lower trust development. Second, variety in the degree and type of prior experience of the partners involved in a dyad can result in differences on what has been learned by partners in the past, which can affect subsequent cooperative dynamics. Lastly, examining how information regarding the reputation of partners influences partner selection and subsequent cooperative performance would also be an interesting avenue for future research.

## Supporting information

S1 Appendix Experimental instructions. (DOC)

S2 Appendix Payoffs for different investment outcomes per game. (DOCX)

S1 Data Experimental data full. (SAV)

S2 Data Experimental data excluding removed observations. (SAV)
