# Supplementary material for: Dynamic influences on cooperation in a social dilemma: How type of experience and communication affect behavioral spillovers
Source: PLoS One. 2019 Mar 12;14(3):e0213038. doi: 10.1371/journal.pone.0213038 (PMC6413919; doi:10.1371/journal.pone.0213038)
Supplement: S1 Appendix — (DOC) [file pone.0213038.s001.doc]

**S1 Appendix. Experimental instructions.**

**Welcome!**

You are participating in a study of economic decision-making.

The decisions that you make will result in earnings for you, with better decisions resulting in more earnings. You personally will be paid based on your experimental earnings in cash at the end of the session.

Please do not communicate with the other participants in this session other than via the program. Communication outside of the program is not allowed. Any participant found breaking this rule will be expelled from the session and will not be paid its earnings. If at any time you have a question about these instructions or something that happens during the session, please raise your hand and we will answer your question privately.

This session is divided into a number of parts. Each part is introduced with an instruction. Please read these instructions carefully.

Imagine that you are a manager in a firm that produces medicines. As a manager you are responsible for investments in your firm’s own production process and in cooperations with other firms.

A decade ago, your firm successfully introduced the medicine “VU-Brand” on the market. The entire production process for this medicine is owned by your firm. Because patents on sole production of the VU-Brand medicine have expired recently, competitors are now introducing similar medicines on the market against competitive prices. By investing in the firm’s production process of the VU-Brand medicine cost savings can be realized and the medicine can be produced more efficient which is necessary to stay competitive.

Because of these changed market conditions, top management has decided to start the development of a new medicine, called the “VU-personalized-medicine”. An important characteristic of this VU-personalized-medicine is its ultimate treatment effectiveness. Each disease and each patient is unique, which makes that some medicines have no effect on some patients care, while the same medicines are considered highly effective for the treatment of other patients. The VU-personalized-medicine provides an objective basis for considering these individual differences and is tailored to individual patient needs. It is therefore a highly effective medicine that is expected to improve medical care services significantly and become very successful . Because of this, the VU-personalized-medicine can be priced very high.

In order to develop and produce this VU-personalized-medicine, your firms need specific knowledge which is not available within your own firm. Successful market introduction of the VU-personalized-medicine is only possible when it is produced in cooperation with another firm (partner firm[[1]](#footnote-2)) and requires a minimum investment in this cooperative production process.

This part is divided into a series of games and each game will consist of a number of rounds. Each round you will control a fixed amount of 150 resources. You will choose if and how many of these resources you wish to invest in cooperative production activities (to develop and produce the VU-personalized-medicine with a partner firm) and how many resources to retain to use internally for individual production activities (to make this internal production process of the VU-Brand medicine more efficient). Each round you have to allocate the full amount (150) of your controlled resources, but in every round you have discretion in how you invest these resources to the individual and cooperative production activities. Your partner controls the same amount of resources and at the same time as you are making your decisions, the manager of your partner firm will be making its decisions. These decisions determine the total value created (in Experimental Euro’s (EE)) for your firm’s shareholders and will influence your own profits, since managerial compensation is linked to performance; the more profits you earn the more money you earn. For this part you will be paid in cash at the end of the session based on your earnings in Experimental Euro’s.

The resources you retain for individual production will determine your profit from individual production activities. These profits will be privately to you. You will receive 1000 Experimental Euros of profit for each unit of resource retained for individual production. The maximum amount of profit per round that you can earn with individual production activities is thus (150 x 1.000 EE) 150.000 EE.

Successful market introduction of the VU-personalized-medicine is only possible when it is produced in cooperation with a partner firm (because of the required specific knowledge) and thus requires that both you and your partner invest in the cooperative production activities. The minimum required investment in this cooperative production process is thus higher than the amount of resources controlled by any one firm alone (150), but you and your partner do not know the exact minimum required investment amount. This makes investing in cooperative production activities more risky than investing in individual production activities, but successful cooperative production activities also result in significant higher profits for both you and your partner.

If you and your partner make the cooperative production activities successful, you and your partner together will receive a fixed bonus of 500.000 EE. This bonus from successful cooperative production activities is always equally shared between you and your partner (250.000 EE to you and 250.000 EE to your partner) and is fixed. If the total amount of resources allocated to cooperative production activities is higher than the minimum required investment, it will not influence the amount of the bonus from cooperative activities. However, if more than the minimum required amount of resources is allocated to cooperative activities, this will yield a total profit of 200 Experimental Euros per resource invested above this minimum required amount. Profits from resource investments above the minimum required amount are also always equally shared between you and your partner (100 EE per resource for you and 100 per resource for your partner). You and your partner will always equally share the total amount of profits from the cooperative production activities, no matter what your respective resources investments in cooperative production activities are. The minimum amount of profit from successful cooperative production activities is thus 250.000 EE, while the maximum amount of profit from individual production activities is (150 x 1.000 EE) 150.000 EE.

When the total amount of resources invested in cooperative production activities is less than the minimum required amount, it will lead to cooperation failure and your invested resources in cooperative production activities are then considered to be lost and will not yield any profits to you. However, in this case your partner is able to profit from your resource investment by appropriating your firm-specific knowledge and this will yield profits privately to him. In this case your partner profits with 500 EE per resource invested by you in failed cooperative production. Vice versa, you will profit with 500 EE per resource invested by your partner in failed cooperative production. These profits are privately to you or your partner.

On the next page you will find an overview of the key characteristics of your task.

- Each round you will control a fixed amount of 150 resources.
- You will always receive 1000 Experimental Euros of profit for each unit of resource retained for individual production. The maximum amount of profit per round that you can earn with individual production activities is thus (150 x 1.000 EE) 150.000 EE.
- Successful cooperative production requires that both you and your partner invest in the cooperative production activities and requires that in total a minimum amount is invested by you and your partner. When the total amount of resources invested in cooperative production activities is less than the minimum required amount, it will lead to cooperation failure.
- When the cooperation fails, your invested resources in cooperative production activities are considered to be lost and will not yield any profits to you. In this case your partner is able to profit from your resource investment and earns 500 EE per resource invested by you in the failed cooperative production. Vice versa, you will profit with 500 EE per resource invested by your partner in failed cooperative production.
- If you and your partner make the cooperative production activities successful, both you and your partner will receive a fixed bonus of 250.000 EE. The minimum amount of profit from successful cooperative production activities is thus 250.000 EE.
- If more than the minimum required amount of resources is invested in cooperative activities, this will yield you a profit of 100 Experimental Euros per resource invested above this minimum required amount. The same applies for your partner. Notice that the bonus for successful cooperative production is fixed, and the investments above the minimum required investment yield 100 EE per resource to you, while investments in individual production activities yield 1.000 EE per resource.

Before you can start with making your investment decisions, you will now get some last instructions.

In every round, you will be facing a screen that looks like this:

At the top of the screen you are reminded what game and round you are in. Below this information, you will be making your resource investment decision. Remember, your decision is how many resources to allocate toward cooperative production activities and how many to keep to use internally for individual production activities. You are free to choose the amount of resources allocated to individual production and cooperative activities in every round. Once you have decided about your investment, click on the button below which says “Invest Resources”. It is not possible to undo or change your investment decisions once you have registered you investment decisions by clicking on this button.

You can use the calculator to make calculations in order to retrieve how and why your investments resulted in the round outcomes. You can also use your calculations document which you can find in your envelope.

When you have made your investment decision, the program will record your decision and checks if the total investment is 150 resources. If not, you will return to the decision entry screen again, otherwise you have to wait for the decision of your partner. The screen for the next round will automatically be displayed when your partner made its decision.

Each round you will have a maximum amount of time to make your decision. You will find a countdown clock which tells you the time you have left to make your decision. If you are not able to make and register your decisions within the given time, the program will automatically record an investment of zero (0) resources to both individual production as cooperative production and you will not earn any profit in that round.

Once you and your partner have made your decisions, the program will calculate your profits for that round. This information is provided in the results table.

You are first given information regarding your own resource investment decision and you are then informed whether the minimum required investment for successful cooperative production was met or not. Then your total round profit is displayed, followed by the profits from individual production. Further, your cumulative earnings for the round are reported and your cumulative earnings for the game. You will not get any information about your partners resource investment and the profit of your partner in a particular round.

Below the results table you will find a chat box. You can use this chat box to communicate with your partner. You can continue to chat with your partner during the entire game.

For each message in the chat box you can access who send that message (you or your partner). You may not identify yourself personally or ask for the identity of your partner. This rule is designed to protect the anonymity of the participants. You will not know the identity of your partner in any round, not even after the end of the session. Any participant found breaking these rules will be expelled from the session.

At the bottom of the screen you are provided with the investment characteristics overview. You can also find this overview on paper in your envelope.

Now is the time for questions. If you have a question about these instructions please raise your hand and we will answer your question privately.

1. We will refer to this partner firm as your partner. [↑](#footnote-ref-2)
