# Supplementary material for: Dynamic influences on cooperation in a social dilemma: How type of experience and communication affect behavioral spillovers
Source: PLoS One. 2019 Mar 12;14(3):e0213038. doi: 10.1371/journal.pone.0213038 (PMC6413919; doi:10.1371/journal.pone.0213038)
Supplement: S2 Appendix — (DOCX) [file pone.0213038.s002.docx]

**S2 Appendix. Payoffs and incentives per game.**

In this appendix, we further delineate the payoff structure of each game in our experiment. In the first game the threshold value is set at 198. First assume that both players invest their 150 resources in cooperative production activities, a strategy we call *‘full cooperation’*. The threshold amount of 198 is met, so both players get the fixed cooperation bonus of 500.000. In addition, they will earn 200 per resource above the threshold, yielding 20.400 (= (300 – 198) x 200). Total cooperative profits of 520400 (500.000 + 20.400) are equally shared among both players, so they both earn 260.200. If only one of the partners is investing all of its resources in the cooperative activity and the other player is investing all of its resource in individual production activities (*‘individualistic’* strategy), this will lead to cooperation failure (due to the threshold of 198 resources). The player who is investing all of its resources in cooperative activities, will not make any profit with its resource allocation. However, the player who is investing all of its resources in individual production activities will not only earn (150 x 1.000 =) 150.000 of profits from individual production activities but will also profit from the cooperative resource allocation of its partner (150 x 500 = 75.000). For this player, total profits will thus be 150.000 + 75.000 = 225.000. This strategy results in game two and game three in exactly the same amount of profit. If both players decide to keep the full amount of resources under their control internally for individual production activities (strategy *‘no cooperation’*), they will both earn only individual profits. Under this scenario, both players will earn (150 x 1.000) = 150.000. In game two and game three, this strategy results in the exactly the same amount of profit. Since both players earn 1.000 per resource invested in individual production activities and 200 in total (100 per player) per resource invested in cooperative production activities above the threshold, both players have no incentive to allocate more resources than the required threshold value for cooperative success. The joint-optimal resource allocation (strategy *‘joint optimal’*) would be both players investing 99 resources in cooperative production activities, which yields 301.000 (= (500.000 / 2) + (51 x 1.000)) of profits for both players. Each player can do even better than this joint optimal outcome. One would prefer that its partner is allocating all of its resources (150) towards the cooperative activities such that only 48 resources (198 – 150) have to be invested in cooperation to make it a success and get the cooperative bonus. The other 102 resources can then for individual production. In this scenario (‘*individual optimal*’), the player who is investing all (150) of its resources in cooperative activities earns 250.000 while the other player earns (250.000 + (102 x 1.000) =) 352.000. Accordingly, although the incentives are aligned with cooperative behavior, as long as the cooperative activities are successful, each player can do even better by free riding on the investment of its partner. If follows that the individual player has an incentive to contribute as little as necessary to make the cooperative activities successful (with a minimum of 48 resources), as this yields the highest individual profit (352.000).

For game two and game three, we only discuss resulting payoffs based on the ‘*full cooperation*’, ‘*joint optimal*’ and ‘*individual optimal*’ strategies. Game two has a threshold value of 158. Under ‘*full* *cooperation*’, both players invest their 150 resources in cooperative production activities. The required threshold value of 158 is met, so they get the fixed cooperation bonus of 500.000. In addition, they will earn 200 per resource above the threshold, yielding ((150 + 150 – 158) x 200) 28.400. Given that total cooperative profits of 528.400 are equally shared, they both earn 264.200. The ‘*joint-optimal*’ resource allocation strategy would be both players investing 79 resources in cooperative production activities, which yields 321.000 of profits (= (500.000 / 2) + (71 x 1.000)) for both players. The ‘*individual optimal*’ strategy, assuming that the partner invests 150 in cooperative production activities, maximizes individuals outcomes given that one only has to invest 8 in cooperative activities to make the cooperation a success and can use the other 142 resources for individual production. With this strategy, the player who is investing all (150) of its resources in cooperative activities earns 250.000 while the other player earns (250.000 + (142 x 1.000) =) 392.000.

In game three, the threshold value is set at 238. When both players invest their 150 resources in cooperative production activities (‘*full cooperation*’), the threshold amount of 238 is met, so they get the fixed cooperation bonus of 500.000 in total. In addition, they will in total earn 200 per resource above the threshold, yielding ((150 + 150 – 238) x 200) 12.400, so they both earn 256.200 (= (500.000 + 12.400) / 2). A threshold of 238 implies that for the ‘*joint-optimal*’ strategy, based on equality, both players need to invest 119 resources in cooperative production activities, which yields 281.000 of profits for both players (= (500.000 / 2) + (31 x 1.000). However, each player would prefer that its partner is allocating all of its resources (150) towards the cooperative activities such that one only has to invest 88 resources (238 – 150) to get the cooperative bonus. This will result in an individual profit of (250.000 + (62 x 1.000)) 312.000, while the other party would earn 250.000.

It follows that the individual player has an incentive to contribute as little as necessary to make the cooperative activities successful (with a minimum of respectively 48, 8 and 88 resources), as this yields the highest individual profit (respectively 352.000, 392.000 and 312.000). However, these payoffs also violate principles of equality and fairness, as the partner only earns 250.000 during these scenarios.

In the table below, we provide an overview of the payoffs based on the different resource allocation strategies for the three experimental games (profit numbers x 1.000).

|  | Game I | | Game II | | Game III | |
| --- | --- | --- | --- | --- | --- | --- |
| Strategy | Player 1 | Player 2 | Player 1 | Player 2 | Player 1 | Player 2 |
| Full cooperation   - Players profit | (0 : 150)  260,20 | (0 : 150)  260,20 | (0 : 150)  264,20 | (0 : 150)  264,20 | (0 : 150)  256,20 | (0 : 150)  256,20 |
| - Total profit | 520,40 | | 528,40 | | 512,40 | |
| Joint optimal   - Players profit | (51 : 99)  301 | (51 : 99)  301 | (71 : 79)  321 | (71 : 79)  321 | (31 : 119)  281 | (31 : 119)  281 |
| - Total profit | 602 | | 642 | | 562 | |
| Individual optimal   - Players profit | (102 : 48)  352 | (0 : 150)  250 | (142 : 8)  392 | (0 : 150)  250 | (62 : 88)  312 | (0 : 150)  250 |
| - Total profit | 602 | | 642 | | 562 | |
| Individualistic   - Players profit | (0 : 150)  0 | (150 : 0)  225 | (0 : 150)  0 | (150 : 0)  225 | (0 : 150)  0 | (150 : 0)  225 |
| - Total profit | 225 | | 225 | | 225 | |
| No cooperation   - Players profit | (150 : 0)  150 | (150 : 0)  150 | (150 : 0)  150 | (150 : 0)  150 | (150 : 0)  150 | (150 : 0)  150 |
| - Total profit | 300 | | 300 | | 300 | |
